# Risk category system to identify pituitary adenoma patients with *AIP* mutations

**DOI:** 10.1136/jmedgenet-2017-104957

**Published:** 2018-02-10

**Authors:** Francisca Caimari, Laura Cristina Hernández-Ramírez, Mary N Dang, Plamena Gabrovska, Donato Iacovazzo, Karen Stals, Sian Ellard, Márta Korbonits

**Affiliations:** 1 Centre of Endocrinology, William Harvey Research Institute, Barts and the London School of Medicine and Dentistry, Queen Mary University of London, London, UK; 2 Department of Endocrinology, Universitat Autònoma de Barcelona (UAB), Barcelona, Spain; 3 Section of Endocrinology and Genetics, Eunice Kennedy Shriver National Institute of Child Health and Human Development (NICHD), National Institutes of Health (NIH), Bethesda, Maryland, USA; 4 Department of Molecular Genetics, Royal Devon and Exeter NHS Foundation Trust, Exeter, UK

**Keywords:** AIP mutations, acromegaly, familial pituitary adenoma, screening, risk category system

## Abstract

**Background:**

Predictive tools to identify patients at risk for gene mutations related to pituitary adenomas are very helpful in clinical practice. We therefore aimed to develop and validate a reliable risk category system for aryl hydrocarbon receptor-interacting protein (*AIP*) mutations in patients with pituitary adenomas.

**Methods:**

An international cohort of 2227 subjects were consecutively recruited between 2007 and 2016, including patients with pituitary adenomas (familial and sporadic) and their relatives. All probands (n=1429) were screened for *AIP* mutations, and those diagnosed with a pituitary adenoma prospectively, as part of their clinical screening (n=24), were excluded from the analysis. Univariate analysis was performed comparing patients with and without *AIP* mutations. Based on a multivariate logistic regression model, six potential factors were identified for the development of a risk category system, classifying the individual risk into low-risk, moderate-risk and high-risk categories. An internal cross-validation test was used to validate the system.

**Results:**

1405 patients had a pituitary tumour, of which 43% had a positive family history, 55.5% had somatotrophinomas and 81.5% presented with macroadenoma. Overall, 134 patients had an *AIP* mutation (9.5%). We identified four independent predictors for the presence of an *AIP* mutation: age of onset providing an odds ratio (OR) of 14.34 for age 0-18 years, family history (OR 10.85), growth hormone excess (OR 9.74) and large tumour size (OR 4.49). In our cohort, 71% of patients were identified as low risk (<5% risk of *AIP* mutation), 9.2% as moderate risk and 20% as high risk (≥20% risk). Excellent discrimination (c-statistic=0.87) and internal validation were achieved.

**Conclusion:**

We propose a user-friendly risk categorisation system that can reliably group patients into high-risk, moderate-risk and low-risk groups for the presence of *AIP* mutations, thus providing guidance in identifying patients at high risk of carrying an *AIP* mutation. This risk score is based on a cohort with high prevalence of *AIP* mutations and should be applied cautiously in other populations.

## Introduction

Pituitary adenomas are relatively common lesions, present in approximately 17% of the general population,^1^ although clinically relevant disease is identified in only around 1:1000 subjects.^2 3^ Most pituitary adenomas are sporadic; however, familial cases are increasingly recognised, representing some 5% of all patients presenting with pituitary adenomas.^4^ Mutations in the aryl hydrocarbon receptor-interacting protein (*AIP*) gene predispose to the development of pituitary adenomas but with a low penetrance (20%–23%).^5–8^ *AIP* mutations can be identified either in the context of familial isolated pituitary adenomas (FIPA), defined by the presence of pituitary adenomas in two or more family members with no other syndromic features, or as simplex cases with a germline mutation but no known family history of the disease. The prevalence of *AIP* mutations is around 20% in FIPA kindreds,^7 8^ while in sporadic pituitary adenomas, the prevalence ranges between 3.6% and 20%,^9 10^ depending on the age group studied.

More than 100 different ‘pathogenic’ or ‘likely pathogenic’^11^ germline *AIP* variants have been described (non-sense, missense, in frame deletion/insertion, segmental duplication, large genomic deletion, frameshift, promoter, start codon and splice-site mutations^8^), while several variants are currently considered as having ‘unknown significance’.^12^

Although all types of pituitary adenomas have been described with germline *AIP* mutations, patients with such mutations typically have young-onset growth hormone (GH)-secreting or GH-secreting and prolactin-secreting tumours with generally poor responsivity to conventional treatment, and aggressive behaviour compared with those with no recognised mutation,^7 8^ often requiring repeated surgery and radiotherapy and therefore needing close surveillance.^7 13 14^

Risk assessment for an *AIP* mutation has important clinical implications, and the genetic screening of family members allows for the identification of those at risk of developing aggressive pituitary adenomas.^8 15 16^ Early diagnosis at a non-invasive stage can potentially lead to a higher chance of effective or curative treatment.^8 15^

There are no formal guidelines defining the criteria for genetic screening pituitary adenoma patients for *AIP* mutations, and currently the clinical decision for screening is based on expert recommendation.^16 17^ Identification of *AIP* mutation-positive patients can lead to the detection of carriers with otherwise unrecognised disease,^8 15^ potentially leading to a better prognosis. Our study aimed to develop and validate a risk category system to stratify patients with isolated pituitary adenomas for their risk of carrying *AIP* mutations. This risk category system was designed to serve as an effective tool to aid clinical decision making and the identification of *AIP* mutation carriers in clinical practice.

## Materials and methods

Two thousand two hundred and twenty-seven subjects were included in our database from February 2007 to June 2016, including 1429 affected subjects with pituitary tumours and 798 unaffected relatives. Subjects were recruited via the FIPA consortium, an international research group. The data collected from medical records related to each individual patient were sent to our group, and the information was checked to confirm that all patients met the inclusion criteria. All subjects included signed the informed consent approved by the local ethics committee.

We included patients who presented with FIPA and patients diagnosed with apparently sporadic pituitary adenomas with disease onset at ≤30 years of age. In addition, we also included referred patients with sporadic adenomas and an age of onset >30 years. Patients with X-linked acrogigantism syndrome (XLAG)^18 19^ and patients who presented with other recognised syndromes such as multiple endocrine neoplasia type 1 or type 4, Carney complex and DICER1 syndrome, were excluded.^20^ These conditions were excluded on the basis of clinical, biochemical and, in some cases, genetic testing, as appropriate. Although patients with XLAG also belong to the FIPA group, their clinical characteristics are so distinct that we felt that they should not be included in this risk prediction analysis. Patients diagnosed prospectively as a result of familial screening of known *AIP* mutations were also excluded from the analysis. Genetic screening for *AIP* mutations was performed using Sanger sequencing and multiplex ligation-dependent probe amplification, as previously described.^8^ Genomic DNA was obtained from blood or saliva samples. The pathogenicity of the detected variants was assessed using the Mutation Taster (http://www.mutationtaster.org/), Anovar^21^ and Variant Effect Predictor (VEP)^22^ in silico prediction programmes. We also included published clinical and experimental data on the previously reported variants. Only pathogenic or likely pathogenic variants were considered as mutations.^11^

### Definition of variables

Patients were identified as affected if they had either (1) a pituitary tumour or (2) pituitary hyperplasia associated with hormone hypersecretion. FIPA was defined by the presence of pituitary adenomas in two or more members of a family with no other associated clinical features. The family history included assessment of all known ‘blood relatives’. The diagnoses were categorised as GH excess (including acromegaly and gigantism, with or without prolactin cosecretion), non-functioning pituitary adenoma (NFPA), prolactinoma, Cushing’s disease and other diagnoses (any other type of functioning pituitary tumour). Macroadenomas were defined by tumour size ≥10 mm. Age of onset was defined as the age at presentation of the first symptom. Pituitary apoplexy was defined by a clinical history of haemorrhage and/or infarction of a pituitary adenoma.

### Statistical analysis

The Shapiro-Wilks test was used to assess Gaussian distribution for continuous variables. Normally distributed variables were expressed as mean and SD and were analysed with the Student’s t-test. Median and IQR were used to describe non-normally distributed variables. These variables were analysed with the Mann-Whitney U test. Qualitative variables were expressed as percentage and analysed with the χ^2^ test to compare two or more groups; P*<*0.05 was taken as significant.

The following clinically relevant variables were included to generate the model: family history of pituitary tumours, gender, age of onset (categorised as ≤18 years, 19–30 years and >30 years old), adenoma type (categorised as tumours secreting GH vs others), adenoma size (categorised as macroadenoma vs microadenoma or hyperplasia) and history of pituitary apoplexy. Interactions between all the studied variables were assessed with the likelihood ratio test. Variable selection was carried out through all possible equations methods, where every potential combination of the independent variables were computed and subsequently evaluated to assess the performance of the possible models.^23^ We selected the final model based on the Akaike information criteria, area under the receiver operating characteristic (ROC) curve and the Hosmer-Lemeshow tests. A logistic regression with the selected variables was performed, and results were expressed with an odds ratio (OR) and its 95% confidence interval (CI).

We arbitrarily categorised the risk of *AIP* mutation into low-risk (<5%), moderate-risk (5%–19%) and high-risk (≥20%) groups. Discrimination of the model was assessed with the c-statistic, and its calibration was assessed comparing observed versus model-derived *AIP* mutation risk and with the Hosmer-Lemeshow test. Finally, we assessed internal validity with a cross-validation procedure for a realistic estimation of the performance of the prediction model. Performance measures included *R^2^* (explained variation of the model). Considering the size of our cohort, we randomly divided the sample in five equal-sized parts, and we calculated the difference between our model and the resampling average. STATA software V.13.1 was used for statistical analysis.

## Results

Out of the 1429 pituitary adenoma patients, 153 carried an *AIP* mutation (10.7%). Out of the 343 relatives of patients with *AIP* mutations, 165 were carriers of an *AIP* mutation (48.1%). The clinical characteristics of the whole cohort are detailed in table 1.

**Table 1:** Clinical characteristics of the whole cohort

Clinical characteristic	n=1405*
*AIP* mutation, n (%)	134 (9.5)
Familial, n (%)	607 (43.2)
Gender, n (% male)	680 (48.5)
Diagnosis, n (%)	
GH excess	767 (55.5)
NFPA	185 (13.4)
Prolactinoma	344 (24.9)
Cushing’s disease	74 (5.4)
Other diagnosis	11 (0.8)
Age of onset (years)	27.1±13.1
Age at diagnosis (years)	30.8±13.4
Macroadenoma, n (%)	977 (81.5)
Extrasellar extension, n (%)	446 (60.1)
Pituitary apoplexy, n (%)	48 (3.9)

*Prospectively diagnosed patients excluded.

NFPA, non-functioning pituitary adenoma.

Twenty-four family members were prospectively diagnosed with a pituitary adenoma, 19 of these carried an *AIP* mutation (clinical characteristics are included in online supplementary table 1), while five belonged to *AIP* mutation-negative families. Prospectively diagnosed patients were excluded from the analysis.

10.1136/jmedgenet-2017-104957.supp1 Supplementary file 1

Six novel *AIP* mutations were found in one patient each, their characteristics are detailed in table 2. All *AIP* mutations identified are listed in online supplementary table 2.

**Table 2:** Novel *AIP* mutations not previously reported. gnomAD: http://gnomad.broadinstitute.org/

AIP mutation	MAF in gnomAD	Variant type	In silico prediction*	Probability score†	Gender	Familial versus simplex	Diagnosis‡	Age at diagnosis	Age at onset
c.240_241delinsTG (p.M80_R81delinsIG)	Not reported	Insertion deletion	High	Disease causing 1	M	Simplex	Gigantism	8	5
c.333delC (p.K112Rfs*44)	Not reported	Frameshift	High	Disease causing 1	F	Simplex	Gigantism	9	7
c.376_377delCA (p.Q126Dfs*3)	Not reported	Frameshift	High	Disease causing 1	F	Simplex	Gigantism	13	10
c.605A>G (p.Y202C) §	Not reported	Missense	High	Disease causing 0.99	M	Simplex	Gigantism	10	10
c.645+1G>C (p.?)	Not reported	Splicing	High	Disease causing 1	M	Simplex	Acromegaly	33	24
c.991T>C (p.331Rext91)	Not reported	Missense	High	Polymorphism 0.99	M	Simplex	Gigantism	16	12

*In silico prediction of probability of damaging mutation by Variant Effect Predictor and Anovar.

†Probability of pathogenic mutation by Mutation Taster.

‡All patients had macroadenoma, and none of them presented with pituitary apoplexy.

§This missense variant affects position 22 in the first tetratricopeptide domain of AIP, a well-conserved position in various tetratricopeptide domain proteins.^32 38^

AIP, aryl hydrocarbon receptor-interacting protein; MAF, minor allele frequency.

The age of onset was significantly lower in *AIP-*positive versus *AIP-*negative patients (16 (14–24) (IQR) vs 25 (19-33) years, P<0.001), as was the age at diagnosis (21 (16–31) vs 29 (22–38) years, P*<*0.001). Table 3 contains the comparison of clinical characteristics of *AIP* mutation-positive and *AIP*-negative patients.

**Table 3:** Clinical characteristics comparing *AIP*-positive and *AIP*-negative patients

Clinical characteristic	*AIP* positive	*AIP* negative	P value
Familial, n (%)	89 (66.4)	518 (40.8)	<0.001
Gender, n (% male)	83 (61.9)	597 (47.1)	0.001
Diagnosis, n (%)			<0.001
GH excess	119 (88.8)	648 (52)	
NFPA	4 (3)	181 (14.5)	
Prolactinoma	11 (8.2)	333 (26.7)	
Cushing’s disease	0	74 (5.9)	
Other diagnosis	0	11 (0.9)	
Age of onset (years and percentages)			<0.001
0–18	79 (60.3)	259 (23.6)	
19–30	33 (25.2)	506 (46)	
>30	19 (14.5)	336 (30.5)	
Age at diagnosis (years and percentages)			<0.001
0–18	53 (40.5)	163 (14.1)	
19–30	44 (33.6)	497 (43)	
>30	34 (26)	495 (42.9)	
Maximum diameter (mm)*	16 (10.7–25)	20 (11–30)	0.518
Macroadenoma, n (%)	112 (93.3)	865 (80.2)	<0.001
Extrasellar extension, n (%)	52 (81.3)	394 (58.1)	<0.001
Pituitary apoplexy, n (%)	12 (9.5)	36 (3.3)	0.001
Number of treatments*	2 (1–3)	1(1–2)	0.055

*Median and IQR.

NFPA, non-functioning pituitary adenoma.

**Figure 1:**
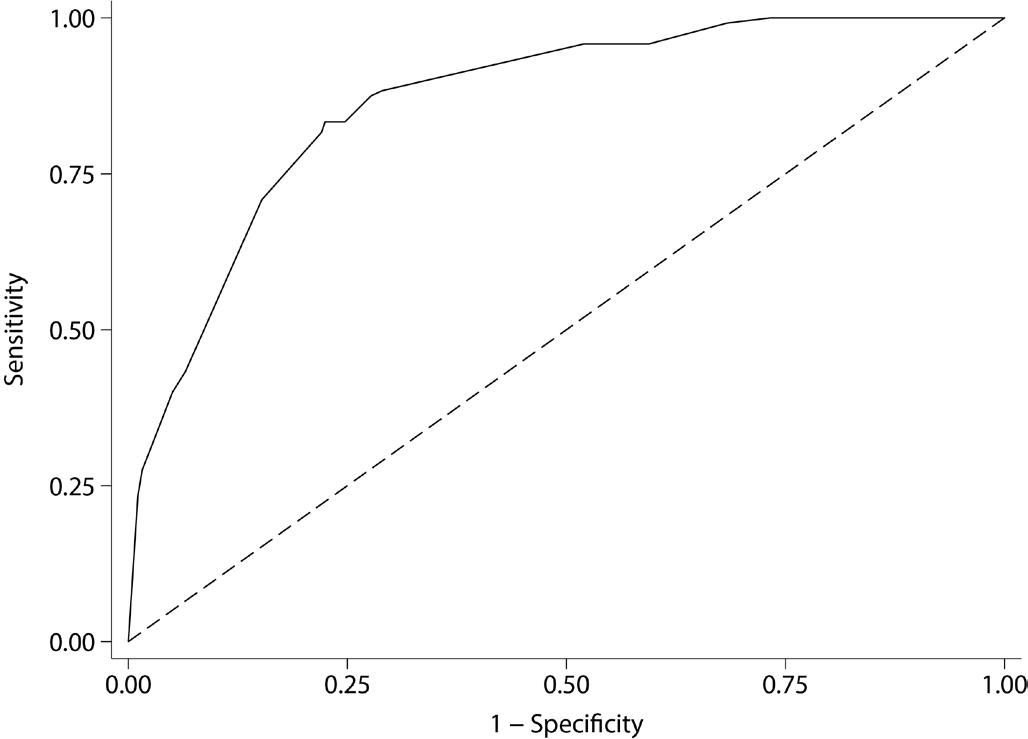
Area under the receiver operating characteristic curve of the *AIP* mutation risk category system is 0.87 (95% CI 0.84 to 0.90), indicating an excellent discriminating power.

**Figure 2:**
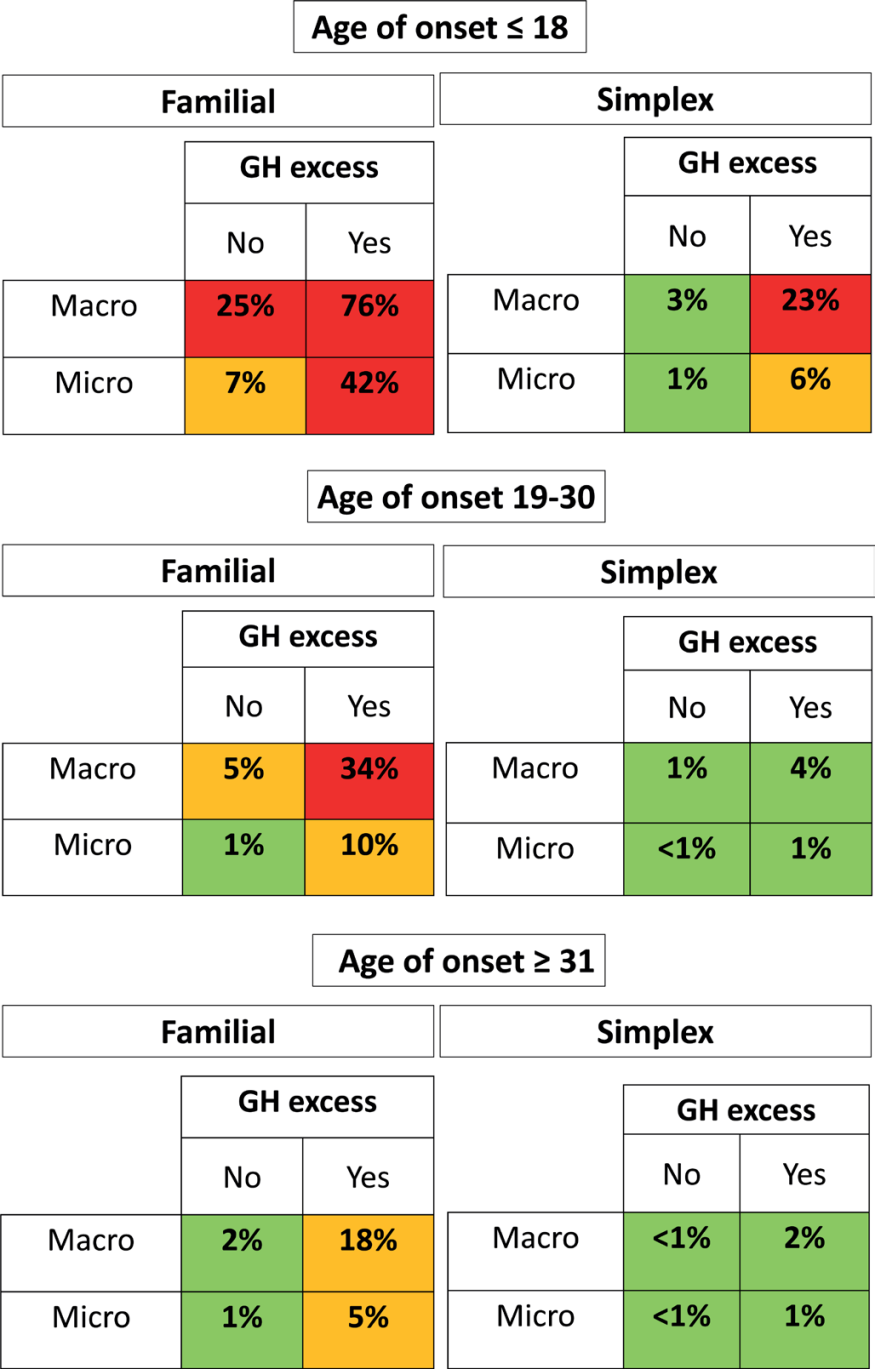
Risk stratification for *AIP* mutations, classified as low risk (<5%), moderate risk (5%–19%) or high risk (≥20%). Red: risk of *AIP* mutation ≥20%; orange: risk of *AIP* mutation between 5% and 19%; green: risk of *AIP* mutation <5%. GH, growth hormone; macro, macroadenoma; micro, microadenoma; simplex, patients with no known family history.

The likelihood ratio test to evaluate interaction terms was non-significant (P*=*0.149), hence interaction terms were excluded from the model. The variable selection process suggested that pituitary apoplexy and gender should be removed from the model, as they did not add predictive power. A markedly increased risk of an *AIP* mutation was associated with having a family history, a GH-excess adenoma, macroadenomas and young age of onset. The variables included in the predictive model are listed in table 4 in the order of their statistical strength for prediction. Good discriminative power was achieved with the area under the curve (AUC), reaching a value of 0.87 (95% CI 0.84 to 0.90) (figure 1). We stratified the risk of having an *AIP* mutation into low risk (<5%), moderate risk (5%–19%) and high risk (≥20%), based on our predictive model. Figure 2 shows stratified risks according to age, family history, tumour type and tumour size. In our cohort, enriched with familial, GH-secreting adenomas and young-onset cases, 70.8% of patients were identified as low risk, 9.2% as intermediate risk, while 20% were at high risk (risk ≥20%). Calibration results, comparing observed and model-predicted *AIP* mutation risk across the three risk groups, are depicted in the online supplementary figure 1. The Hosmer-Lemeshow test was non-significant (P*=*0.213), suggesting that the model is well calibrated.

**Table 4:** Logistic regression to generate a predictive model for *AIP* mutations*

Variable	OR (95% CI)	P value
Age of onset		
>30	1	–
0–18	14.34 (7.41 to 29.31)	<0.001
19–30	2.26 (1.17 to 4.35)	0.015
Positive family history	10.85 (6.48 to 18.16)	<0.001
Diagnosis		
Others	1	–
GH excess	9.74 (5.12 to 18.52)	<0.001
Size (macroadenoma)	4.49 (1.91 to 10.59)	0.001

*Variables are listed in the order of their statistical strength for prediction and each OR is adjusted for all the other variables.

AIP, aryl hydrocarbon receptor-interacting protein; GH, growth hormone.

Finally, the model showed good internal validation, as tested by the cross-validation technique, as the R^2^ shrinkage was <10% in absolute terms (from 0.294 to 0.223).

## Discussion

Using state-of-the-art statistical methods applied to a large cohort of patients, we have identified four significant predictors for the presence of carrying an *AIP* mutation, and these are immediately accessible for routine clinical practice. Our data suggest that a positive family history, young age of onset, somatotroph tumour type and large tumour size can predict the risk of an *AIP* mutation according to our risk model, which has been validated in a large series of patients. Once a mutation carrier is identified, genetic testing can be performed for family members. The overall risk category of a kindred should be based on the risk score of the family member with the highest risk.

Despite the increasing number of genes associated with FIPA,^5 18^ formal guidelines do not currently include recommendations for screening for *AIP* mutations,^24 25^ and therefore such screening is usually performed on the basis of expert recommendations.^16 17 26 27^ These recommendations include patients who have (1) a family history of pituitary adenoma, (2) childhood-onset pituitary adenoma or (3) a pituitary somatotroph or lactotroph macroadenoma diagnosed before the age of 30 years; however, no data stratifying the different risks between these groups have been provided.^9 10 16 17^ Here we provide risk stratification for *AIP* mutation positive patients, using a combination of clinical variables, all of which should be easily available at the time of diagnosis.

Not surprisingly, all the variables included in our *AIP* risk category system have been repeatedly reported as typical clinical features of *AIP* mutation-positive patients. The age of disease onset is the strongest predictive factor, with a maximum risk for an *AIP* mutation present in those patients who presented with an adenoma during childhood (OR 14.3 (95% CI 7.4 to 27.7), P*<*0.001). This result was expected, as the prevalence of *AIP* mutations in paediatric cases has been reported to be between 6% and 23%.^9 10 28 29^ An age between 19 and 30 years (OR 2.3 (95% CI 1.2 to 4.4), P*=*0.015) is also a strong predictor. In a previous study, among subjects with sporadic macroadenomas diagnosed before the age of 30 years, an *AIP* mutation was found in 11.7% overall, with a positive finding in 13.3% of patients with somatotrophinomas, 11.5% of those with prolactinomas and 6.3% of those with NFPAs.^28^ Taking the historical 10% risk cut-off for genetic screening,^30^ patients with familial GH-secreting macrodenoma, ≤30-year-old patients with familial GH-secreting microadenomas and sporadic childhood-onset GH-secreting macroadenomas are all above this threshold.

The second strongest predictive variable was positive family history of pituitary adenomas (OR 10.85 (95% CI 6.48 to 18.16), P*<*0.001). Although the majority of FIPA families have not yet had the causative gene identified, the largest available cohorts found that about 20% of FIPA kindreds harbour a heterozygous germline mutation in the *AIP* gene,^7 8^ with the overall rate being slightly higher in homogeneous versus heterogeneous kindreds (22.8% and 16.7%, respectively).^16^

GH excess is also a good independent predictor for *AIP* mutations in our model (OR 9.74 (95% CI 5.12 to 18.52), P*<*0.001). One of the most important characteristics of patients with *AIP* mutations is the predominance of somatotrophinomas or somatolactotrophinomas, which account for around 80% of the cases. Prolactinomas and clinically NFPAs with positive GH and/or PRL immunostaining are also well described, while ACTH-secreting or TSH-secreting adenomas or gonadotrophin positive or null cell NFPAs are very rare.^31^

In addition, we demonstrate that patients with macroadenomas have more than four times the risk of harbouring an *AIP* mutation compared with those with microadenomas (OR 4.49 (95% CI 1.91 to 10.59), P*=*0.001). Patients with *AIP* mutations have macroadenomas in up to 90% of the cases, and their tumours are significantly larger and more frequently show an extrasellar extension compared with non-mutated familial^8^ and sporadic cases.^6–8^ Cases of double adenomas have also been described among *AIP* mutation positive patients,^32^ while pituitary hyperplasia associated with GH excess^33 34^ is rare.

We also evaluated other clinical characteristics as possible predictors of *AIP* mutations. For instance, *AIP* mutation-positive patients frequently have a history of pituitary apoplexy, which is often the presenting feature.^8 33 35^ It is unclear whether this is due to the fact that these tumours are large and rapidly growing adenomas or whether an additional molecular mechanism renders these adenomas prone to apoplexy. In our study, pituitary apoplexy was significantly more frequent in *AIP* mutated tumours than in negative cases (9.5% vs 3.3%, P*=*0.001); however, this variable did not add any predictive power to the risk model when we adjusted for the other variables.

Gender distribution was statistically significant in the univariate analysis (61.9% vs 47.1% males, P*=*0.001), but not when adjusted for the other variables. There is no clear consensus in the published literature about the gender distribution of *AIP* mutation-positive patients. While several studies reported an increased prevalence of male patients, ascertainment bias probably plays a role, as in large *AIP* mutation positive families the percentage of affected male patients was lower compared with sporadic *AIP* mutation-positive cases.^8^

Using the described model, we were able to stratify the risk of *AIP* mutation into low, moderate and high categories, and we believe this system can be an easy-to-use tool in clinical practice. The model performs well in terms of discrimination, calibration and internal validation. AUC was 0.87 (95% CI 0.84 to 0.90), where 0.5 represents no discrimination and 1 represents perfect discrimination, indicating that our model achieved an excellent discriminatory power.^36^ Additionally, there were no obvious differences between observed and model-predicted *AIP*-positive patients. The Hosmer-Lemeshow test showed adequate goodness of fit of our model.^36^ We have validated our model using an internal cross-validation procedure, one of the preferable methods when external validation is not feasible.^36^ The performance of the model was evaluated comparing the explained variation of the model (*R* ^2^) in each of the five equal samples of the data and the total sample, achieving a reduction of *R* ^2^ <10%.^36^ To the best of our knowledge, there is no other available *AIP* risk category system assessment in the literature for comparison.

Using our risk stratification model, we are able to: (1) describe the risk factors of carrying an *AIP* mutation; (2) quantify the predictive value for each risk factor adjusted by the others; and (3) estimate the individual risk of carrying an *AIP* mutation for a given patient. We expect this tool to be valuable for clinicians to improve the decision-making process of referring patients for genetic screening based on the individual risk of *AIP* mutation.

**Figure 3:**
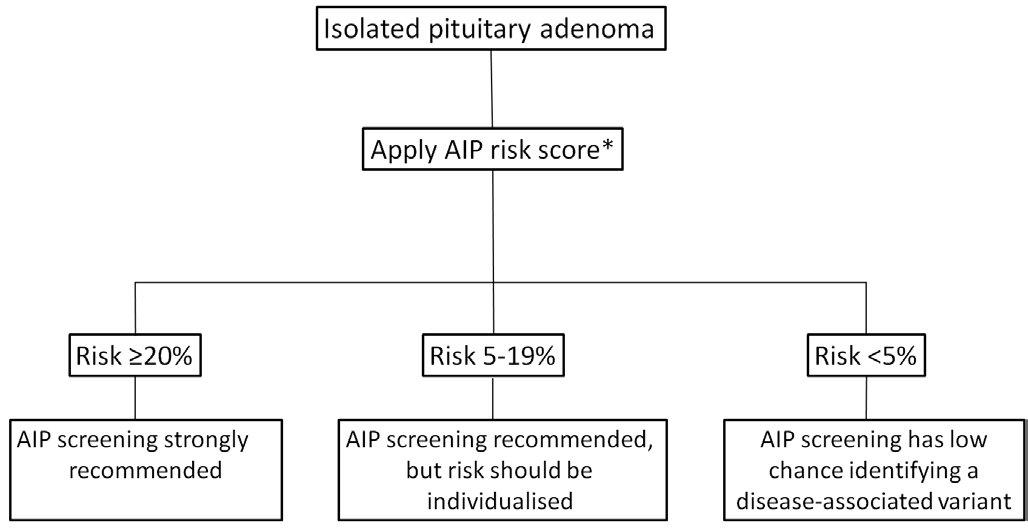
AIP screening algorithm based on the proposed risk category system. The overall risk category of a kindred should be based on the risk score of the family member with the highest risk. AIP, aryl hydrocarbon receptor-interacting protein. *See figure 2.

A screening algorithm based on the results of our risk category system is shown in figure 3. We need to emphasise that we used age of onset and not age of diagnosis for this analysis. This parameter is often more subjective and needs careful history taking, reviewing parents’ height and available photographic evidence of change of features. Patients with pituitary gigantism should be considered to have childhood-onset disease and offered screening.

There are some limitations inherent to our risk model. First, our risk score is based on a cohort enriched with familial, young-onset patients and GH-excess tumours as the number of *AIP* mutated patients in unselected cohorts is low.^9 37^ Although all possible diagnostic groups have some representation in our study, caution should be taken when extrapolating these results to a population with significantly different prevalence of *AIP* mutations than the one found in our cohort; this score ideally estimates the risk in patients where the mutation is already suspected. Second, the determination of age of onset can be subjective and subject to patient recall. Nevertheless, when comparing the model using the age of onset with the one produced using age of diagnosis, the AUC was significantly better using age of onset rather than age at diagnosis; this might be explained due to the well-documented delay of diagnosis in patients with acromegaly. To minimise subjectivity, we categorised the variable age of onset into three broad groups. Finally, it was not possible to perform an external validation of the model due to the relatively low number of cases with *AIP* mutations in our cohort (although it is the largest *AIP* mutation positive series so far described), which precluded splitting the sample into a derivation and validation group. However, we have successfully validated the model using an internal cross-validation method.

In summary, the risk category system we have developed has the potential for widespread use as it includes readily available predictors. We believe this tool, ideally used in patients where the mutation is already suspected, can facilitate the most effective use of genetic screening, which we believe is currently clearly underused, allowing the identification of patients who carry *AIP* mutations and providing the opportunity of early diagnosis in at-risk relatives.
