# Supplementary material for: Risk category system to identify pituitary adenoma patients with AIP mutations
Source: J Med Genet. 2018 Feb 10;55(4):254–60. doi: 10.1136/jmedgenet-2017-104957 (PMC5869708; doi:10.1136/jmedgenet-2017-104957)
Supplement: Supplementary file 1 [file jmedgenet-2017-104957supp001.docx]

**Supplementary Table 1. Clinical characteristics of the *AIP* positive prospectively diagnosed patients**

| Clinical characteristic | N=19 |
| --- | --- |
| Familial, n (%) | 19 (100%) |
| Gender, n (% male) | 12 (63.2%) |
| Diagnosis, n (%)  GH excess  NFPA | 9 (47.4%)  10 (52.6%) |
| Age at diagnosis (years) | 30 [19-37] |
| Maximum diameter (mm)* | 6 [4.9-10] |
| Macroadenoma, n (%) | 6 (31.6 %) |
| Extrasellar extension, n (%) | 2 (11.8%) |
| Pituitary apoplexy, n (%) | 0 (0%) |
| Number of treatments* | 0 [0-2] |

NFPA: non-functioning pituitary adenoma

*Median and interquartile range

**Supplementary Table 2. List of *AI****P* **mutations in our cohort divided into truncating and non-truncating**

| Truncating mutations | Non-truncating mutations † |
| --- | --- |
| g.4856_4857CG>AA[1–3] | c.140_163del (p.G47_R54del)[4] |
| c.1-?_993+?del- (whole gene deletion)[1] | c.469-2A>G (p.E158_Q184del)[5–7] |
| c.100-1025_279+357del (p.A34_K93del) (exon 2 deletion)[8] | c.562C>T(p.R188W)[9] |
| c.240_241delinsTG (p.M80_R81delinsIG) | **c.605A>G (p.Y202C)** |
| c.241C>T (p.R81*)[2,3,10–12] | c.713G>A (p.C238Y)[3,13] |
| c.249G>T (p.G83Afs*15)[1] | c.760T>C (p.C254R)[9] |
| c.333delC (p.K112Rfs*44) | c.762C>G (p.C254W)[9] |
| c.338_341dupACCC (p.L115Pfs*16)[14,15] | c.805_825dup (p.F269_H275dup)[2,3,6] |
| c.376_377delCA (p.Q126Dfs*3) | c.807C>T (p.(=))[1,3,8,16–18] |
| c.3G>A (p.?)[19] | c.811C>T p.R271W[1,4,17,20] |
| c.40C>T (p.Q14*)[14,21–23] | c.815G>G (p.G272D)[19,24] |
| c.427C>T (p.Q143*)[14] | c.872_877delTGCTGG (p.V291_L292del)[25] |
| c.490C>T (p.Q164*)[1] | **c.991T>C(p.331Rext91)** |
| c.570C>G (p.Y190*)[14] |  |
| c.645+1G>C (p.?) |  |
| c.662dupC (p.E222*)[1] |  |
| c.70G>T (p.E24*)[3,13] |  |
| c.74_81delins7 (p.L25Pfs*130)[1,26] |  |
| c.783C>G (p.Y261*)[6,14,27] |  |
| c.787+9C>T[14] |  |
| c.804C>A (p.Y268*)[10,14,28] |  |
| c.816delC (p.K273Rfs*30)[14] |  |
| c.868A>T (p.K290*)[14] |  |
| c.910C>T (p.R304*)[3–6,17,21,27,29] |  |
| c.967delC (p.R323Gfs*39)[14] |  |
| c.976_977insC (p.G326Afs*?)[14] |  |
| c.978dupG (p.I327Dfs*?)[14] |  |

Mutations in bold are novel mutations not previously described. These patients’ clinical characteristics are shown in Table 1.

† Patients with the c.911G>A (p.R304Q) and c.100-18C>T variants were excluded from this study, as recent data suggest that these might represent variants of unknown significance.

**Supplemental Figure 1. Observed versus model-derived *AIP* mutation risk model with low (<5%), moderate (5-19%) and high risk (≥20%) categories.**


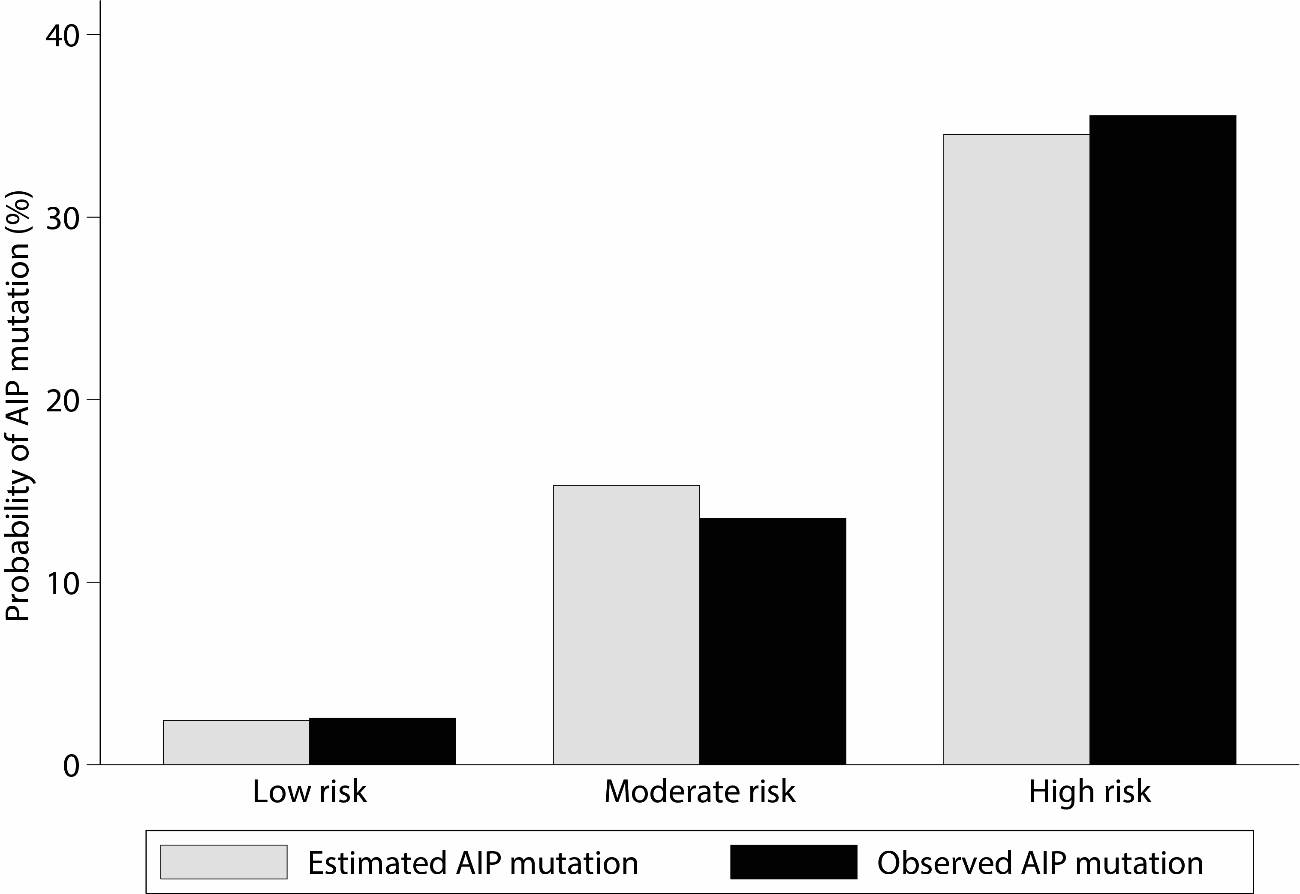


The similar probabilities for estimated and observed risk indicates a good calibration of the model.

**REFERENCES SUPPLEMENTARY MATERIAL**

1. Igreja S, Chahal HS, King P, Bolger GB, Srirangalingam U, Guasti L, Chapple JP, Trivellin G, Gueorguiev M, Guegan K, Stals K, Khoo B, Kumar A V, Ellard S, Grossman AB, Korbonits M. Characterization of aryl hydrocarbon receptor interacting protein (AIP) mutations in familial isolated pituitary adenoma families. *Hum Mutat.* 2010;31(8):950–60.

2. Soares BS, Eguchi K, Frohman LA. Tumor deletion mapping on chromosome 11q13 in eight families with isolated familial somatotropinoma and in 15 sporadic somatotropinomas. *J Clin Endocrinol Metab.* 2005;90(12):6580–7.

3. Leontiou CA, Gueorguiev M, van der Spuy J, Quinton R, Lolli F, Hassan S, Chahal HS, Igreja SC, Jordan S, Rowe J, Stolbrink M, Christian HC, Wray J, Bishop-Bailey D, Berney DM, Wass J a H, Popovic V, Ribeiro-Oliveira A, Gadelha MR, Monson JP, Akker S a, Davis JRE, Clayton RN, Yoshimoto K, Iwata T, Matsuno A, Eguchi K, Musat M, Flanagan D, Peters G, Bolger GB, Chapple JP, Frohman L a, Grossman AB, Korbonits M. The role of the aryl hydrocarbon receptor-interacting protein gene in familial and sporadic pituitary adenomas. *J Clin Endocrinol Metab.* 2008;93(6):2390–401.

4. Daly AF, Vanbellinghen J-F, Khoo SK, Jaffrain-Rea M-L, Naves LA, Guitelman MA, Murat A, Emy P, Gimenez-Roqueplo A-P, Tamburrano G, Raverot G, Barlier A, De Herder W, Penfornis A, Ciccarelli E, Estour B, Lecomte P, Gatta B, Chabre O, Sabaté MI, Bertagna X, Garcia Basavilbaso N, Stalldecker G, Colao A, Ferolla P, Wémeau J-L, Caron P, Sadoul J-L, Oneto A, Archambeaud F, Calender A, Sinilnikova O, Montañana CF, Cavagnini F, Hana V, Solano A, Delettieres D, Luccio-Camelo DC, Basso A, Rohmer V, Brue T, Bours V, Teh BT, Beckers A. Aryl hydrocarbon receptor-interacting protein gene mutations in familial isolated pituitary adenomas: analysis in 73 families. J *Clin Endocrinol Metab.* 2007;92(5):1891–6.

5. Cazabat L, Libè R, Perlemoine K, René-Corail F, Burnichon N, Gimenez-Roqueplo AP, Dupasquier-Fediaevsky L, Bertagna X, Clauser E, Chanson P, Bertherat J, Raffin-Sanson ML. Germline inactivating mutations of the aryl hydrocarbon receptor-interacting protein gene in a large cohort of sporadic acromegaly: Mutations are found in a subset of young patients with macroadenomas. *Eur J Endocrinol.* 2007;157(1):1–8.

6. Cazabat L, Bouligand J, Salenave S, Bernier M, Gaillard S, Parker F, Young J, Guiochon-Mantel A, Chanson P. Germline AIP mutations in apparently sporadic pituitary adenomas: Prevalence in a prospective single-center cohort of 443 patients. J *Clin Endocrinol Metab*. 2012;97(4):663–70.

7. Martucci F, Trivellin G, Korbonits M. Familial isolated pituitary adenomas: An emerging clinical entity. *J Endocrinol Invest.* 2012 Dec 27;35(11):1003–14.

8. Georgitsi M, De Menis E, Cannavò S, Mäkinen MJ, Tuppurainen K, Pauletto P, Curtò L, Weil RJ, Paschke R, Zielinski G, Wasik A, Lubinski J, Vahteristo P, Karhu A, Aaltonen L a. Aryl hydrocarbon receptor interacting protein (AIP) gene mutation analysis in children and adolescents with sporadic pituitary adenomas. *Clin Endocrinol (Oxf).* 2008;69(4):621–7.

9. Hernández-Ramírez LC, Martucci F, Morgan RML, Trivellin G, Tilley D, Ramos-Guajardo N, Iacovazzo D, D’Acquisto F, Prodromou C, Korbonits M. Rapid Proteasomal Degradation of Mutant Proteins Is the Primary Mechanism Leading to Tumorigenesis in Patients With Missense AIP Mutations. *J Clin Endocrinol Metab.* 2016;101(8):3144–54.

10. Toledo R a, Lourenço DM, Liberman B, Cunha-Neto MBC, Cavalcanti MG, Moyses CB, Toledo SP a, Dahia PLM. Germline mutation in the aryl hydrocarbon receptor interacting protein gene in familial somatotropinoma. *J Clin Endocrinol Metab.* 2007;92(5):1934–7.

11. Guaraldi F, Corazzini V, Gallia GL, Grottoli S, Stals K, Dalantaeva N, Frohman LA, Korbonits M, Salvatori R. Genetic analysis in a patient presenting with meningioma and familial isolated pituitary adenoma (FIPA) reveals selective involvement of the R81X mutation of the AIP gene in the pathogenesis of the pituitary tumor. *Pituitary*. 2012 Dec 13;15(Suppl 1):61–7.

12. Luccio-Camelo DC, Une KN, Ferreira RES, Khoo SK, Nickolov R, Bronstein MD, Vaisman M, Teh BT, Frohman LA, Mendonça BB, Gadelha MR. A meiotic recombination in a new isolated familial somatotropinoma kindred. *Eur J Endocrinol.* 2004 May;150(5):643–8.

13. Gadelha MR, Prezant TR, Une KN, Glick RP, Moskal SF, Vaisman M, Melmed S, Kineman RD, Frohman LA. Loss of heterozygosity on chromosome 11q13 in two families with acromegaly/gigantism is independent of mutations of the multiple endocrine neoplasia type I gene. *J Clin Endocrinol Metab.* 1999 Jan;84(1):249–56.

14. Hernández-Ramírez LC, Gabrovska P, Dénes J, Stals K, Trivellin G, Tilley D, Ferraù F, Evanson J, Ellard S, Grossman AB, Roncaroli F, Gadelha MR, Korbonits M. Landscape of familial isolated and young-onset pituitary adenomas: prospective diagnosis in AIP mutation carriers. *J Clin Endocrinol Metab.* 2015;100(9):E1242–54.

15. Stratakis CA, Tichomirowa MA, Boikos S, Azevedo MF, Lodish M, Martari M, Verma S, Daly AF, Raygada M, Keil MF, Papademetriou J, Drori-Herishanu L, Horvath A, Tsang KM, Nesterova M, Franklin S, Vanbellinghen J-F, Bours V, Salvatori R, Beckers A. The role of germline AIP, MEN1, PRKAR1A, CDKN1B and CDKN2C mutations in causing pituitary adenomas in a large cohort of children, adolescents, and patients with genetic syndromes. *Clin Genet.* 2010;78(5):457–63.

16. McCarthy MI, Noonan K, Wass JAH, Monson JP. Familial acromegaly: studies in three families. *Clin Endocrinol (Oxf)*. 1990 Jun;32(6):719–28.

17. Tichomirowa M a., Barlier A, Daly AF, Jaffrain-Rea ML, Ronchi C, Yaneva M, Urban JD, Petrossians P, Elenkova A, Tabarin A, Desailloud R, Maiter D, Schürmeyer T, Cozzi R, Theodoropoulou M, Sievers C, Bernabeu I, Naves L a., Chabre O, Montañana CF, Hana V, Halaby G, Delemer B, Labarta Aizpún JI, Sonnet E, Ferrandez Longás Á, Hagelstein MT, Caron P, Stalla GK, Bours V, Zacharieva S, Spada A, Brue T, Beckers A. High prevalence of AIP gene mutations following focused screening in young patients with sporadic pituitary macroadenomas. *Eur J Endocrinol.* 2011;165(4):509–15.

18. Oriola J, Lucas T, Halperin I, Mora M, Perales MJ, Alvarez-Escolá C, Paz DM-N, Díaz Soto G, Salinas I, Julián MT, Olaizola I, Bernabeu I, Marazuela M, Puig-Domingo M. Germline mutations of AIP gene in somatotropinomas resistant to somatostatin analogues. *Eur J Endocrinol.* 2013;168(1):9–13.

19. Radian S, Diekmann Y, Gabrovska P, Holland B, Bradley L, Wallace H, Stals K, Bussell A-M, McGurren K, Cuesta M, Ryan AW, Herincs M, Hernández-Ramírez LC, Holland A, Samuels J, Aflorei ED, Barry S, Dénes J, Pernicova I, Stiles CE, Trivellin G, McCloskey R, Ajzensztejn M, Abid N, Akker SA, Mercado M, Cohen M, Thakker R V, Baldeweg S, Barkan A, Musat M, Levy M, Orme SM, Unterländer M, Burger J, Kumar A V, Ellard S, McPartlin J, McManus R, Linden GJ, Atkinson B, Balding DJ, Agha A, Thompson CJ, Hunter SJ, Thomas MG, Morrison PJ, Korbonits M. Increased Population Risk of AIP-Related Acromegaly and Gigantism in Ireland. *Hum Mutat.* 2017;38(1):78–85.

20. Jennings JE, Georgitsi M, Holdaway I, Daly AF, Tichomirowa M, Beckers A, Aaltonen LA, Karhu A, Cameron FJ. Aggressive pituitary adenomas occurring in young patients in a large Polynesian kindred with a germline R271W mutation in the AIP gene. *Eur J Endocrinol.* 2009 Nov 1;161(5):799–804.

21. Vierimaa O, Georgitsi M, Lehtonen R, Vahteristo P, Kokko A, Raitila A, Tuppurainen K, Ebeling TML, Salmela PI, Paschke R, Gündogdu S, De Menis E, Mäkinen MJ, Launonen V, Karhu A, Aaltonen LA. Pituitary adenoma predisposition caused by germline mutations in the AIP gene. *Science*. 2006;312(5777):1228–30.

22. Georgitsi M, Raitila A, Karhu A, Tuppurainen K, Mäkinen MJ, Vierimaa O, Paschke R, Saeger W, van der Luijt RB, Sane T, Robledo M, De Menis E, Weil RJ, Wasik A, Zielinski G, Lucewicz O, Lubinski J, Launonen V, Vahteristo P, Aaltonen LA. Molecular diagnosis of pituitary adenoma predisposition caused by aryl hydrocarbon receptor-interacting protein gene mutations. *Proc Natl Acad Sci U S A*. 2007;104(10):4101–5.

23. Raitila A, Georgitsi M, Karhu A, Tuppurainen K, Mäkinen MJ, Birkenkamp-Demtröder K, Salmenkivi K, Ørntoft TF, Arola J, Launonen V, Vahteristo P, Aaltonen LA. No evidence of somatic aryl hydrocarbon receptor interacting protein mutations in sporadic endocrine neoplasia. *Endocr Relat Cancer.* 2007 Sep 1;14(3):901–6.

24. Karaca Z, Taheri S, Tanriverdi F, Unluhizarci K, Kelestimur F. Prevalence of AIP mutations in a series of Turkish acromegalic patients: are synonymous AIP mutations relevant? *Pituitary*. 2015;18(6):831–7.

25. Ramírez-Rentería C, Hernández-Ramírez LC, Portocarrero-Ortiz L, Vargas G, Melgar V, Espinosa E, Espinosa-de-los-Monteros AL, Sosa E, González B, Zúñiga S, Unterländer M, Burger J, Stals K, Bussell A-M, Ellard S, Dang M, Iacovazzo D, Kapur S, Gabrovska P, Radian S, Roncaroli F, Korbonits M, Mercado M. AIP mutations in young patients with acromegaly and the Tampico Giant: the Mexican experience. *Endocrine.* 2016;53(2):402–11.

26. Jones MK, Evans PJ, Jones IR, Thomas JP. Familial acromegaly. *Clin Endocrinol (Oxf).* 1984 Aug;20(3):355–8.

27. Cuny T, Pertuit M, Sahnoun-Fathallah M, Daly A, Occhi G, Odou MF, Tabarin A, Nunes ML, Delemer B, Rohmer V, Desailloud R, Kerlan V, Chabre O, Sadoul JL, Cogne M, Caron P, Cortet-Rudelli C, Lienhardt A, Raingeard I, Guedj AM, Brue T, Beckers A, Weryha G, Enjalbert A, Barlier A. Genetic analysis in young patients with sporadic pituitary macroadenomas: Besides AIP don’t forget MEN1 genetic analysis. *Eur J Endocrinol.* 2013;168(4):533–41.

28. Jorge BH, Agarwal SK, Lando VS, Salvatori R, Barbero RR, Abelin N, Levine MA, Marx SJ, Toledo SP. Study of the multiple endocrine neoplasia type 1, growth hormone-releasing hormone receptor, Gs alpha, and Gi2 alpha genes in isolated familial acromegaly. *J Clin Endocrinol Metab.* 2001 Feb;86(2):542–4.

29. Occhi G, Trivellin G, Ceccato F, De Lazzari P, Giorgi G, Demattè S, Grimaldi F, Castello R, Davì M V., Arnaldi G, Salviati L, Opocher G, Mantero F, Scaroni C. Prevalence of AIP mutations in a large series of sporadic Italian acromegalic patients and evaluation of CDKN1B status in acromegalic patients with multiple endocrine neoplasia. *Eur J Endocrinol.* 2010;163(3):369–76.
